# Origin of the propatagium in non-avian dinosaurs

**DOI:** 10.1186/s40851-023-00204-x

**Published:** 2023-02-23

**Authors:** Yurika Uno, Tatsuya Hirasawa

**Affiliations:** Department of Earth and Planetary Science, Graduate School of Science, the University of Tokyo, 7-3-1 Hongo, Bunkyo-ku, Tokyo, 113-0033 Japan

## Abstract

Avian wings as organs for aerial locomotion are furnished with a highly specialized musculoskeletal system compared with the forelimbs of other tetrapod vertebrates. Among the specializations, the propatagium, which accompanies a skeletal muscle spanning between the shoulder and wrist on the leading edge of the wing, represents an evolutionary novelty established at a certain point in the lineage toward crown birds. However, because of the rarity of soft-tissue preservation in the fossil record, the evolutionary origin of the avian propatagium has remained elusive. Here we focus on articulated skeletons in the fossil record to show that angles of elbow joints in fossils are indicators of the propatagium in extant lineages of diapsids (crown birds and non-dinosaurian diapsids), and then use this relationship to narrow down the phylogenetic position acquiring the propatagium to the common ancestor of maniraptorans. Our analyses support the hypothesis that the preserved propatagium-like soft tissues in non-avian theropod dinosaurs (oviraptorosaurian *Caudipteryx* and dromaeosaurian *Microraptor*) are homologous with the avian propatagium, and indicate that all maniraptoran dinosaurs likely possessed the propatagium even before the origin of flight. On the other hand, the preserved angles of wrist joints in non-avian theropods are significantly greater than those in birds, suggesting that the avian interlocking wing-folding mechanism involving the ulna and radius had not fully evolved in non-avian theropods. Our study underscores that the avian wing was acquired through modifications of preexisting structures including the feather and propatagium.

**Supplementary Information:**

The online version contains supplementary material available at 10.1186/s40851-023-00204-x.

## Background

Birds possess a suite of characteristics prerequisite for powered flight. The fossil record suggests that the evolutionary processes leading to avian characteristics such as the feather [1, 2], pulmonary air-sac system [3], brain shape [4], and small genome size [5], had proceeded in non-avian dinosaurs, namely, prior to the origin of powered flight.

The avian wing evolved through modification of the forelimb of bipedal theropod dinosaurs. Evolutionary changes in forelimb skeletal morphology had occurred not only in the early evolution of the wing (i.e., among *Archaeopteryx* and more crownward stem birds) but had also accumulated along the non-avian grade of the phylogeny, particularly in non-avian paravians [6]. In this evolutionary process, the morphology of the wrist joint has attracted attention in paleontology, and previous studies revealed that the birds inherited the large flexion of the wrist joint from non-avian theropods [7–10], although the configuration of carpal skeletal elements at the wrist joint had not been conserved during theropod evolution [11, 12]. On the other hand, the orientation of the glenoid on the scapulocoracoid changed after the origin of the avian wing, eventually reducing the energy required for the muscles around the shoulder joint during flapping by the function of the acrocoracohumeral ligament [13]. These skeletal aspects of theropod forelimb evolution imply that the basic skeletal design necessary for the early evolution of powered flight had gradually acquired in both non-avian and stem avian theropod grades.

Although the arrangement of the skeletal muscles changed during the evolution of the avian wing, homologous muscles are mostly identifiable between birds and the other extant diapsids [14–17]. Indeed, the basal theropod *Tawa hallae* likely possessed most of the skeletal muscles on the forelimb seen in birds [17]. However, there is an exception: the propatagial muscle.

**Fig. 1:**
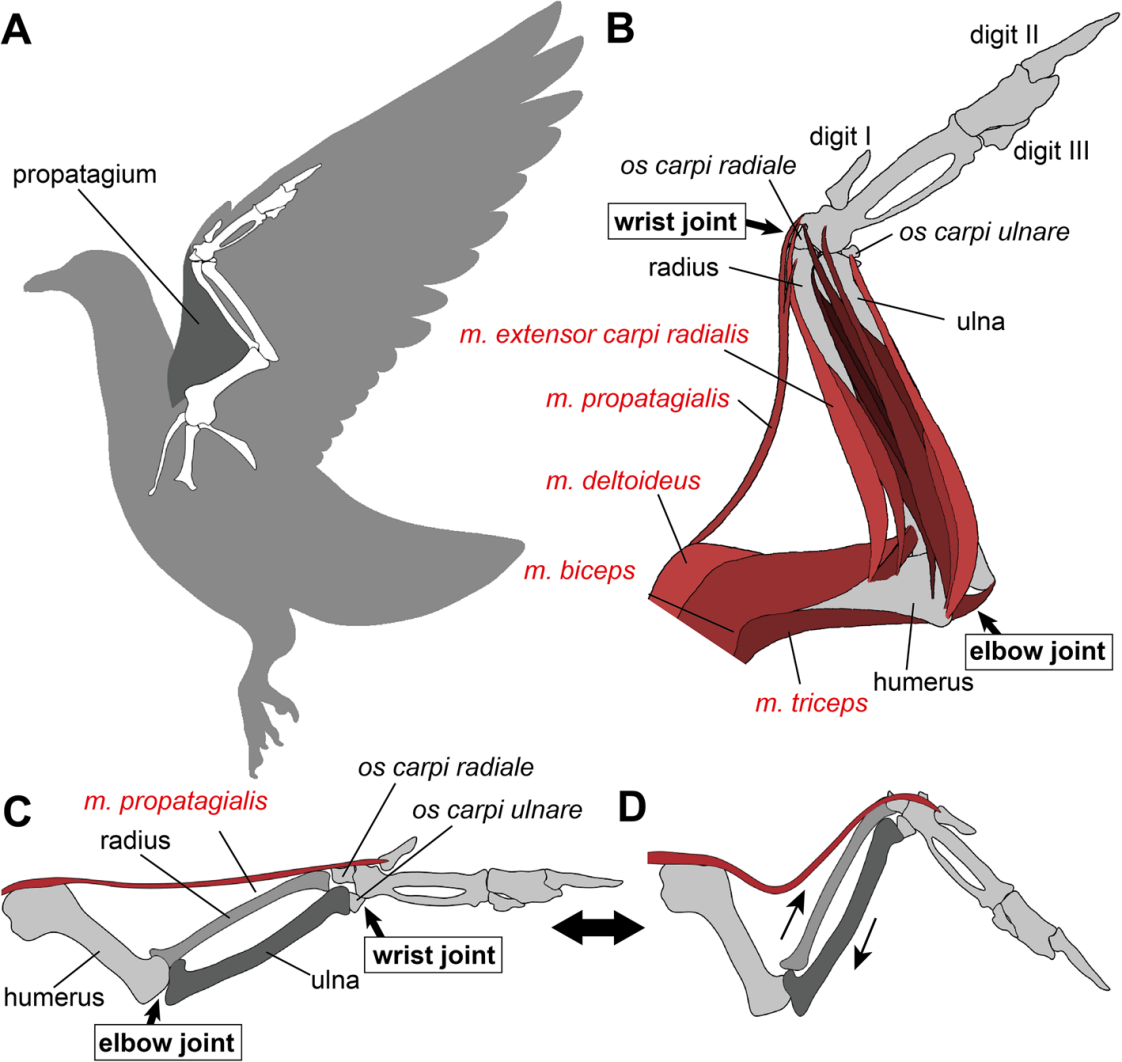
Musculoskeletal system of the avian left wing in ventral view. **A**, **B**, Propatagium (**A**) and forelimb muscles (**B**). **C**, **D**, Synchronous actions between the elbow and wrist joints, at an extension via the function of the *musculus (m.) propatagialis* (**C**) and at a flexion via the interlocking wing-folding system (**D**)

Within the propatagium (Fig. 1A) of the avian wing, the propatagial muscle (*musculus propatagialis*) spans between the shoulder girdle and wrist (Fig. 1B) [14–16, 18]. This structure constitutes the leading edge of the wing and represents an evolutionary novelty in birds. In some species of birds, most of the propatagial muscle is “tendinous” for most of its length, thus has often been called the “propatagial tendon” [19, 20].

In extant birds, at the extension of the elbow joint, the propatagial muscle/tendon synchronously causes the extension of the wrist joint, while keeping the elbow joint at an angle (Fig. 1C) [19–21]. In addition to this structure, extant birds have an interlocking wing-folding mechanism, in which the sliding movement of the ulna and radius causes the folding of the wrist at the flexion of the elbow joint (Fig. 1D) [19, 20]. These synchronized actions of the elbow and wrist joints facilitate the control of wing flapping with only a small volume of muscles in the distal part of the wing. On the other hand, ratites as non-volant birds lack the propatagial structure [14, 22, 23], being consistent with its functional relationship to specific movements of forelimbs.

**Fig. 2:**
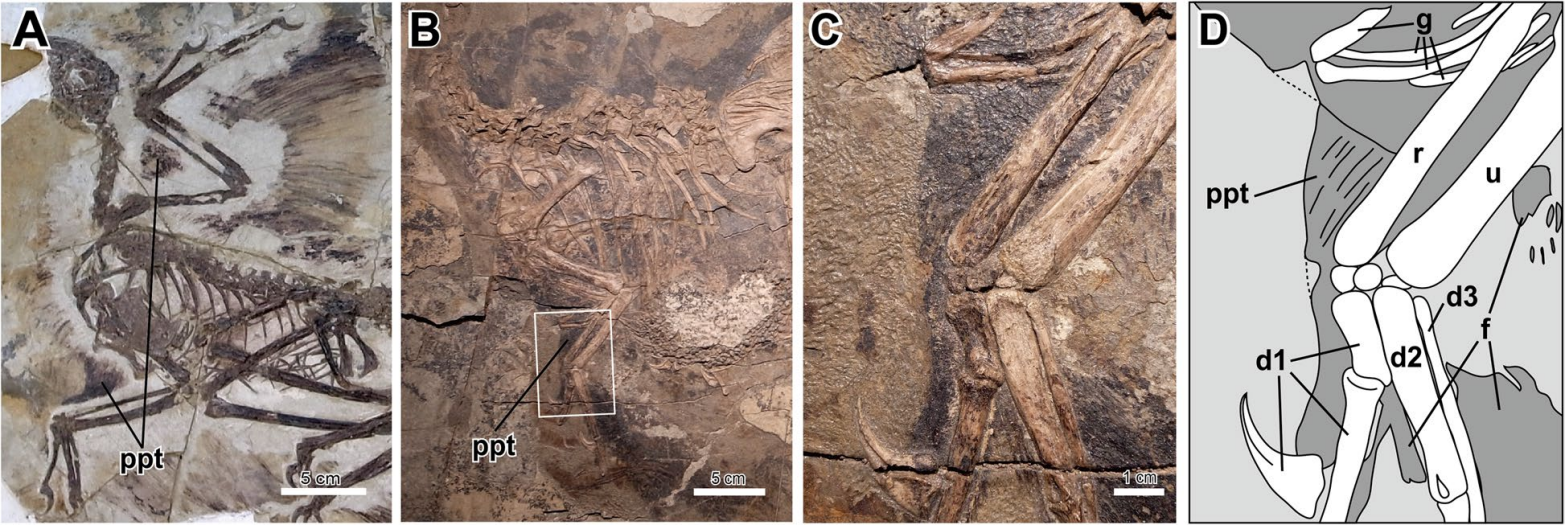
Soft-tissue preservations of putative propatagia in non-avian theropods. **A** *Microraptor gui* (IVPP V 13352). **B**–**D**, *Caudipteryx* sp. (IVPP V 12430). **C** and **D** represent the enlarged image and line drawing of the area of the white box in **B**, respectively. Broken lines in **D** indicate missing borders of the soft tissues. d1–3, digits 1–3; f, feather; g, gastralium; ppt, propatagium; r, radius; u, ulna

From this functional point of view, some studies have discussed the evolutionary origin of the propatagium in the lineage towards birds [10, 24–27]. Although soft-tissue preservation of putative propatagia has been reported for two non-avian theropod species—*Microraptor gui* (Fig. 2A) [28] and *Caudipteryx* sp. (Fig. 2B) [29]—such evidence is absent from some other non-avian theropods with soft-tissue preservation (e.g., *Sinosauropteryx* [30]). However, considering the rarity of soft-tissue preservation, information regarding the early evolution of the propatagium is currently very limited. Also, it may be difficult to identify an osteological correlate, or a muscle attachment site, of the propatagial muscle in fossil skeletons, because this muscle is thin, unlike the other major wing muscles. Therefore, it is substantially impractical to infer the evolution of the propatagial muscle based only on typical morphological analyses.

Unlike soft-tissue preservation, the vertebrate fossil record is relatively rich in preserved articulated skeletons, and the preserved postures of those articulated fossils may reflect morphologies of soft tissues [10, 31]. Because of the restricted movements through the propatagial muscle and skeletal interlocking wing-folding mechanism in wings of extant birds (Fig. 1C, D), the angles of these joints preserved in fossils are expected to fall within a certain range. In particular, the elbow joint in species with a propatagium should not be extended beyond a certain angle in articulated fossil skeletons, because the propatagium restricts the range of the elbow-joint angle in life (Fig. 1C). On the other hand, it is likely that the angles of elbow and wrist joints would be preserved at random in fossils of species devoid of the propatagial structure. Indeed, these postulates on joint angles preserved in articulated skeletons have been addressed in discussions about the evolution of the propatagium in some previous studies [24, 27]. However, there is ample room for analyses of the relationship between the propatagium and preserved posture, particularly in the birds with a propatagium, before reconstructing the evolutionary process.

In this study, we first tested our prediction about the relationship between the propatagium and preserved posture, using fossils of Cenozoic crown-group birds possessing the propatagium and Mesozoic–Cenozoic non-dinosaurian diapsids lacking the propatagium, and demonstrated that the angle of the elbow joint preserved in an articulated fossil skeleton is an indicator of the presence or absence of the propatagium. Second, we measured the joint angles in articulated fossil skeletons of Mesozoic theropods (both non-avian and stem avian) to infer the evolutionary process of the propatagium and the skeletal interlocking wing-folding mechanism.

## Methods

### Criteria of selection of “articulated” fossil specimens

In this study, we collected data about the joint angles in articulated fossil skeletons from previously published images (Table S1). Here, our subjects for measurement were specimens in which all joints of the forelimb are completely articulated: we did not examine fossils wherein only an elbow or a wrist articulation was preserved. When multiple specimens were available for a species, we used a mean value of each joint angle as representative for the species. For an individual with the articulations of both (right and left) forelimbs preserved, the larger value was used as representative for the individual, because the angle of the elbow joint is expected not to exceed a certain value in the presence of the propatagium in our prediction. Angles of the elbow and wrist joints of fossils were measured in ImageJ 1.52a software (https://imagej.nih.gov/ij/) using the “Angle” tool.

For the fossil specimens of *Microraptor gui* (IVPP V 13352) and *Caudipteryx* sp. (IVPP V 12430), direct observations were also conducted for this study.

### Comparative analyses of joint angles preserved in fossils of extant lineages

First, we measured the preserved joint angles of the forelimbs in fossils of crown birds from the Cenozoic, which unequivocally possessed the propatagium, and in fossils of non-dinosaurian diapsids (i.e., a paraphyletic category) from the Mesozoic and Cenozoic (Table S2). Because the latter taxa belonged to the lineages leading toward the extant groups of non-avian diapsids, they serve as representatives lacking the propatagial muscle, while possessing the other forelimb muscles present in the crown birds.

### Trends in preserved joint angles in non-avian theropod phylogeny

Next, we measured the elbow- and wrist-joint angles preserved in articulated fossil skeletons of the Mesozoic theropods (Table S2). Here, to examine large-scale taphonomic trends rather than species-level trends, non-avian theropods were divided into six taxonomic categories (grades): non-dinosaurian diapsids (C0), non-coelurosaurian theropods (C1), non-maniraptoran coelurosaurs (C2), non-paravian maniraptorans (C3), non-avialan paravians (C4), non-pygostylian avialans (C5), pygostylians (C6), and crown birds (C7). To evaluate differences in angles among taxonomic categories, we conducted one-way analysis of variance (ANOVA) in RStudio (2021.09.1 Build 372) (https://www.rstudio.com). To check for any significance in the difference between neighboring taxonomic categories, as well as crown birds and non-dinosaurian diapsids, we performed the Wilcoxon rank sum test in RStudio.

In addition, to evaluate the trends in preserved joint angles along the phylogeny, which was based on the topologies of previous analyses [32–41], parsimony-based ancestral state reconstructions for continuous characters were performed using Mesquite v.3.70 [42].

## Results

### Relationship between the presence of the propatagium and the joint angles preserved in fossils

**Fig. 3:**
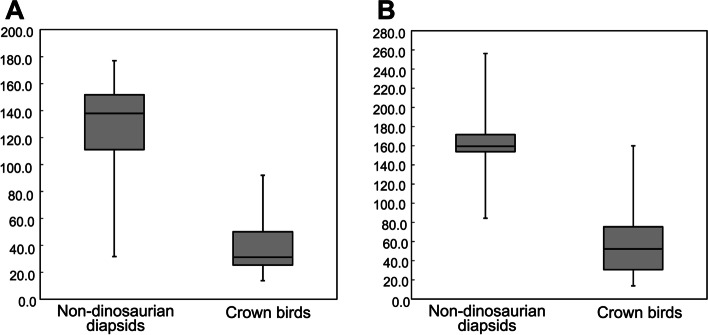
Comparison of preserved elbow and wrist-joint angles between the non-dinosaurian diapsids and crown birds. **A** Elbow-joint angles. **B** Wrist-joint angles. Measurements were obtained from 71 non-dinosaurian diapsid and 35 crown bird articulated fossil skeletons

Comparisons between elbow-joint angles of the crown birds (*n* = 35) and non-dinosaurian diapsids (*n* = 71) showed a significant difference (*p* < 0.001). The median values of elbow angles in the non-dinosaurian diapsids and crown birds were 137.9 and 31.3 degrees, respectively (Fig. 3A). Although the entire ranges of elbow-joint angles of these two categories overlapped at 31.7–92.0 degrees, the interquartile ranges were separated: the interquartile range for the crown birds fell below 50.2 degrees, whereas that for the non-dinosaurian diapsids fell above 111.0 degrees (Fig. 3A). The 95% confidence intervals (mean ± 1.96SE) of the elbow-joint angles were 131.0 ± 8.2 degrees for the non-dinosaurian diapsids and 40.2 ± 8.4 degrees for the crown birds.

In addition, there was a significant difference between the angles of the wrist joints in these two categories (*p* < 0.001). The median values of the wrist-joint angles in the non-dinosaurian diapsids and crown birds were 159.5 and 52.2 degrees, respectively (Fig. 3B).

Accordingly, the elbow- and wrist-joint angles preserved in fossil skeletons differ significantly (*p* < 0.001) between these two taxonomic categories, those possessing and those lacking the propatagium, respectively. As expected, the joint angles of the crown birds were smaller than those of the non-dinosaurian diapsids.

### Changes in joint angles preserved in articulated fossil skeletons in Mesozoic theropod lineage

Among non-avian theropods, a significant difference in average elbow-joint angle was detected by one-way ANOVA (*p* < 0.001). We therefore performed the Wilcoxon rank sum test to test for significant differences between neighboring taxonomic categories in phylogenetic order. The median values of elbow-joint angles in the six categories (C1–C6) between the non-dinosaurian diapsids (C0) and crown birds (C7) are shown in Table 1. A significant difference in elbow-joint angle was detected between C3 (non-paravian maniraptorans) and C4 (non-avialan paravians) (*p* < 0.05).

**Table 1:** Median values of elbow- and wrist-joint angles

	C0	C1	C2	C3	C4	C5	C6	C7
Elbow angle	137.9	88.7	91.3	84.6	36.2	38.0	31.6	31.3
Wrist angle	159.5	112.0	150.3	128.1	36.2	116.3	60.8	52.2

Also, to observe the trends in preserved joint angles in the Mesozoic theropod lineage without focusing on taxonomic categories (C1–C6), we mapped the measured elbow-joint angles onto the phylogeny, and conducted an ancestral state reconstruction based on maximum parsimony (Fig. S1). The result showed that elbow-joint angles decreased along the roots of the Maniraptora and Paraves, thereby being consistent with the statistical analyses of C1–C6. Elbow-joint angles remained small (< 110 degrees) among the Maniraptora.

The averages of wrist-joint angles were also significantly different among taxonomic categories C0–C7, according to one-way ANOVA (*p* < 0.001). The median values of wrist-joint angles are shown in Table 1. The Wilcoxon rank sum test detected a significant difference between C0 (non-dinosaurian diapsids) and C1 (non-coelurosaurian theropods), between C1 and C2 (non-maniraptoran coelurosaurs), and between C5 (non-pygostylian avialans) and C6 (pygostylians) (*p* < 0.05).

With the measured wrist-joint angles mapped onto the phylogeny, the ancestral state reconstruction showed that the preserved wrist-joint angles tended to decrease along the phylogeny almost concomitant with the decreases in the preserved elbow-joint angles (Fig. S2). However, there is a difference in the phylogenetic positions that eventually show joint angles comparable to those of the crown birds: the preserved elbow-joint angles became small in the Maniraptora, whereas the preserved wrist-joint angles decreased greatly after the divergence of *Jeholornis*.

Our direct observations of the fossil specimens of *Microraptor gui* (IVPP V 13352) and *Caudipteryx* sp. (IVPP V 12430) confirmed that soft tissues comparable to the propatagia are preserved on their forelimbs (Fig. 2). In IVPP V 13352 (*M. gui*) the putative propatagia are separated from the other soft tissues (Fig. 2A). On the other hand, the boundary between the soft tissue surrounding the forelimb and that surrounding the trunk is unclear in IVPP V 12430 (*C.* sp.) (Fig. 2B). However, our detailed observation revealed that the soft tissue surrounding the first digit is contiguous with a soft tissue being endowed with a fibrous structure spread wide from the radial side of the forelimb (Fig. 2C, D). Thus, at least the soft tissue spreading from the forearm is distinguishable from the other parts and likely represent the soft tissue comparable to the propatagium, although the possibility that a part of the soft tissue along the upper arm corresponds to the trunk tissues cannot be excluded.

## Discussion

In this study, we analyzed modes of preservation of forelimb postures in articulated fossils. Since each species is mostly represented by a single fossil specimen preserving only a variable posture within the range allowed by the soft-tissue anatomy, species-level macroevolutionary trends are out of the scope of our analyses. Nevertheless, origins of evolutionary novelties of soft tissue including the propatagium can change allowable ranges of preserved posture, and such transitions affect taphonomic trends along phylogeny at higher taxonomic level.

Statistical analyses demonstrated that elbow-joint angles preserved in fossils of crown birds are smaller than those preserved in fossils of non-dinosaurian diapsids. The presence of the propatagium in the crown birds represents the major difference between these two categories. We can exclude the possibility that the skeletal interlocking wing (forelimb)-folding mechanism seen in the crown birds was associated with the difference in elbow-joint angle, because the decreases in angles of the elbow and wrist joints along the Mesozoic theropod phylogeny preserved in fossils were not synchronous. In other words, in some “intermediate” forms between the non-dinosaurian diapsids and crown birds, the elbow and wrist joints were likely not interlocked via the ulna and radius, while the elbow-joint angle remained small. Therefore, the angles of elbow joints preserved in articulated fossil skeletons should be an indicator of the presence or absence of the propatagium.

Based on this relationship between the presence of the propatagium and the elbow-joint angles preserved in fossils, the presence of the propatagium can be inferred in non-avian dinosaurs, which are phylogenetically bracketed by the crown birds and non-dinosaurian diapsids, even if there is no indication of soft tissue (propatagium) in the fossils. It can be safely said that species with preserved elbow-joint angles within the interquartile range of non-dinosaurian diapsids (i.e., larger than 111.0 degrees) did not possess the propatagium.

**Fig. 4:**
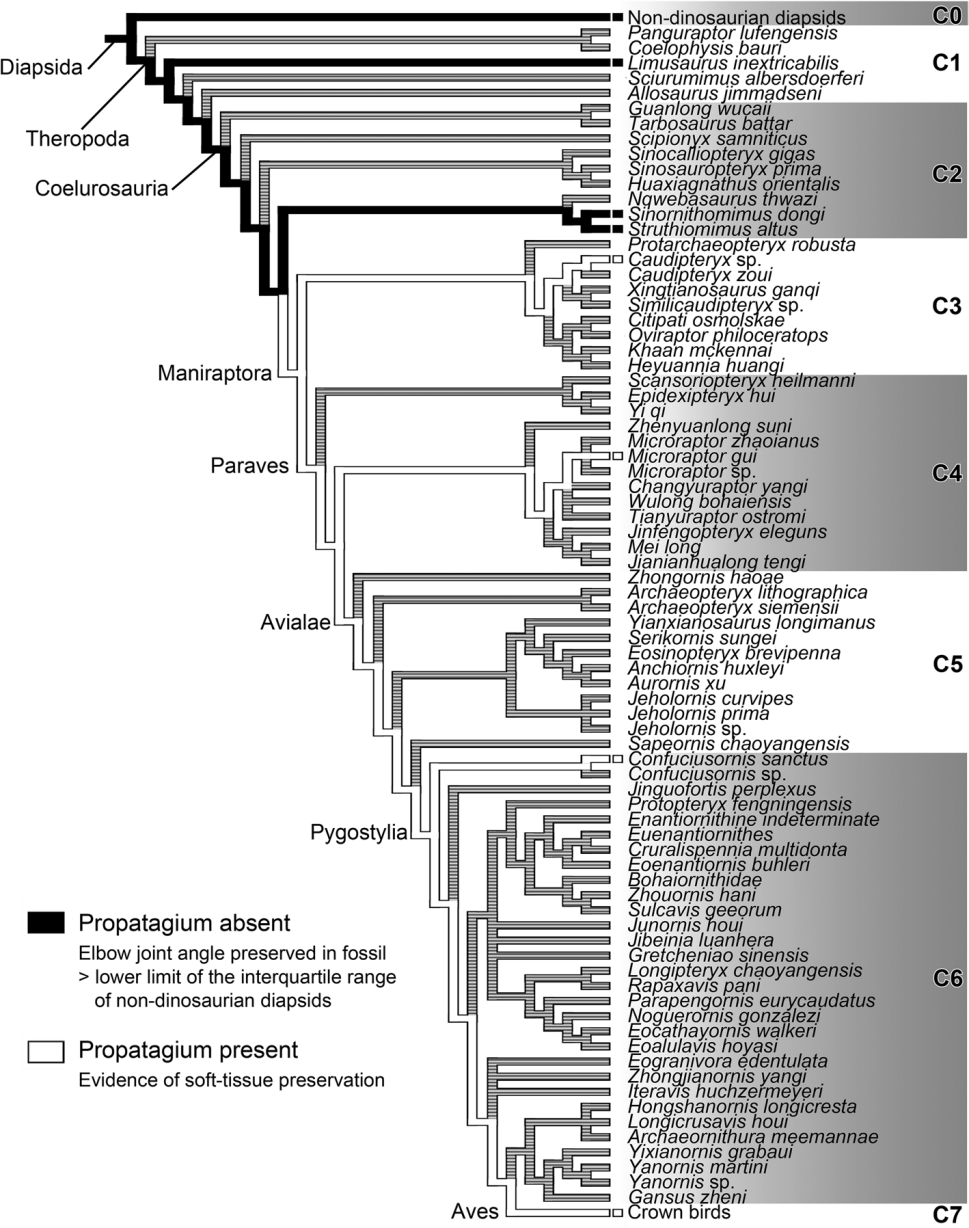
Evolutionary process of the propatagium. The evolutionary process of the propatagium was inferred based on the elbow-joint angles preserved in fossils (evidence for absence) and soft-tissue preservation (evidence for presence)

On the basis of this indicator, we mapped the fossil taxa lacking the propatagium onto the phylogenetic framework, along with the taxa showing soft-tissue preservations of putative propatagia (*Caudipteryx* sp. and *Microraptor gui*) (Fig. 2), and conducted an ancestral state reconstruction (Fig. 4). The result demonstrated that the propatagium had not evolved in non-maniraptoran theropods (C1), whereas it is not inconsistent with the scenario that the Maniraptora globally possessed the propatagium. Since the range of preserved elbow-joint angles began to change at the origin of the Maniraptora (Fig. S1), it is likely that the evolutionary origin of the propatagium dates back to the origin of Maniraptora, and that the propatagia of *Caudipteryx* sp. and *Microraptor gui* were homologous (even in the strict sense) with the avian propatagium. Our analyses also detected a decrease in range of preserved elbow-joint angles in the lineage towards the Paraves, suggesting the propatagium became highly developed in the Paraves.

**Fig. 5:**
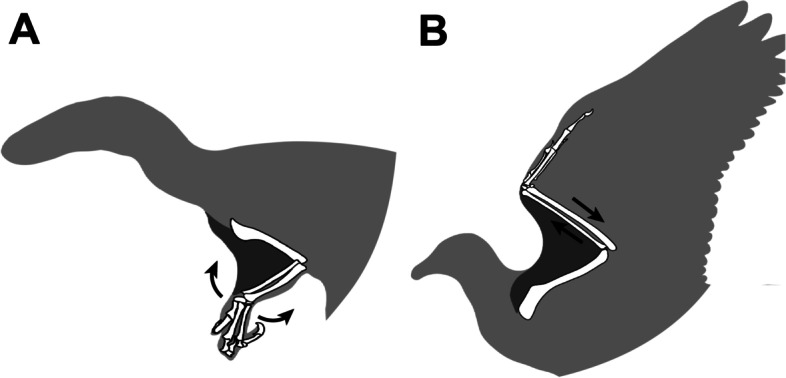
The “intermediate” and avian-state morphotypes. **A** *Deinonychus antirrhopus* representing the “intermediate” morphotype. The propatagium was present, while the wrist could extend toward the radial side in a grasping movement. **B** *Sapeornis chaoyangensis* with the avian interlocking wing-folding system

Among non-avian theropods, the range of preserved angles of the elbow and wrist joints decreased at different points along the phylogeny (Figs. S1, S2). The range of preserved wrist-joint angles are large compared to those of the crown birds in non-pygostylian maniraptorans, which likely possessed the propatagium (Figs. S1, S2). This mode of preservation seen in non-pygostylian maniraptorans (C4, 5) can be explained by the possibility that these species extended the wrist joint toward the radial side when grasping items such as prey. In this scenario, the skeletal interlocking wing (forelimb)-folding system seen in the crown birds was absent in this “intermediate” morphotype possessing the propatagium (Fig. 5), and first evolved among the Pygostylia, in which the grasping capability was not required. This interpretation is consistent with a previous study on the evolution of the wrist skeletal morphology suggesting that the same skeletal architecture of the wrist as in the crown birds first evolved in the clade more crownward than *Sapeornis* [11].

## Conclusions

In this study, we demonstrated that elbow-joint angles preserved in articulated fossil skeletons can be indicators of the presence or absence of the propatagium. Based on this relationship, it is likely that the propatagium first evolved within the Maniraptora. On the other hand, the decrease in preserved wrist-joint angle was observed at a phylogenetic position more derived than that of the decrease in elbow-joint angle, suggesting an “intermediate” morphotype possessing the propatagium on forelimbs used for grasping, rather than for flying.

## Supplementary Information

**Additional file 1.**

## Data Availability

All data obtained in this study were included in the Additional file 1. All specimens are deposited at the institutions listed.

## Abbreviations

C0–7: taxonomic categories 0–7
IVPP: Institute of Vertebrate Paleontology and Paleoanthropology, Beijing, China
