# Supplementary material for: Origin of the propatagium in non-avian dinosaurs
Source: Zoological Lett. 2023 Feb 23;9:4. doi: 10.1186/s40851-023-00204-x (PMC9951497; doi:10.1186/s40851-023-00204-x)
Supplement: Supplementary file 1 — Additional file 1. [file 40851_2023_204_MOESM1_ESM.pdf]

Additional File 1

Origin of the propatagium in non-avian dinosaurs

Yurika Uno and Tatsuya Hirasawa*

Department of Earth and Planetary Science, Graduate School of Science, the University of Tokyo, Tokyo, Japan

*Correspondence: hirasawa@eps.s.u-tokyo.ac.jp

Table of contents

Figure S1. Ancestral state reconstruction of the preserved elbow-joint angles. 2

Figure S2. Ancestral state reconstruction of the preserved wrist-joint angles. 3

Table S1. List of specimens. 4

Table S2. Measurements of the elbow- and wrist-joint angles. 11

List of Institutional Abbreviations 16

Supplementary References 18

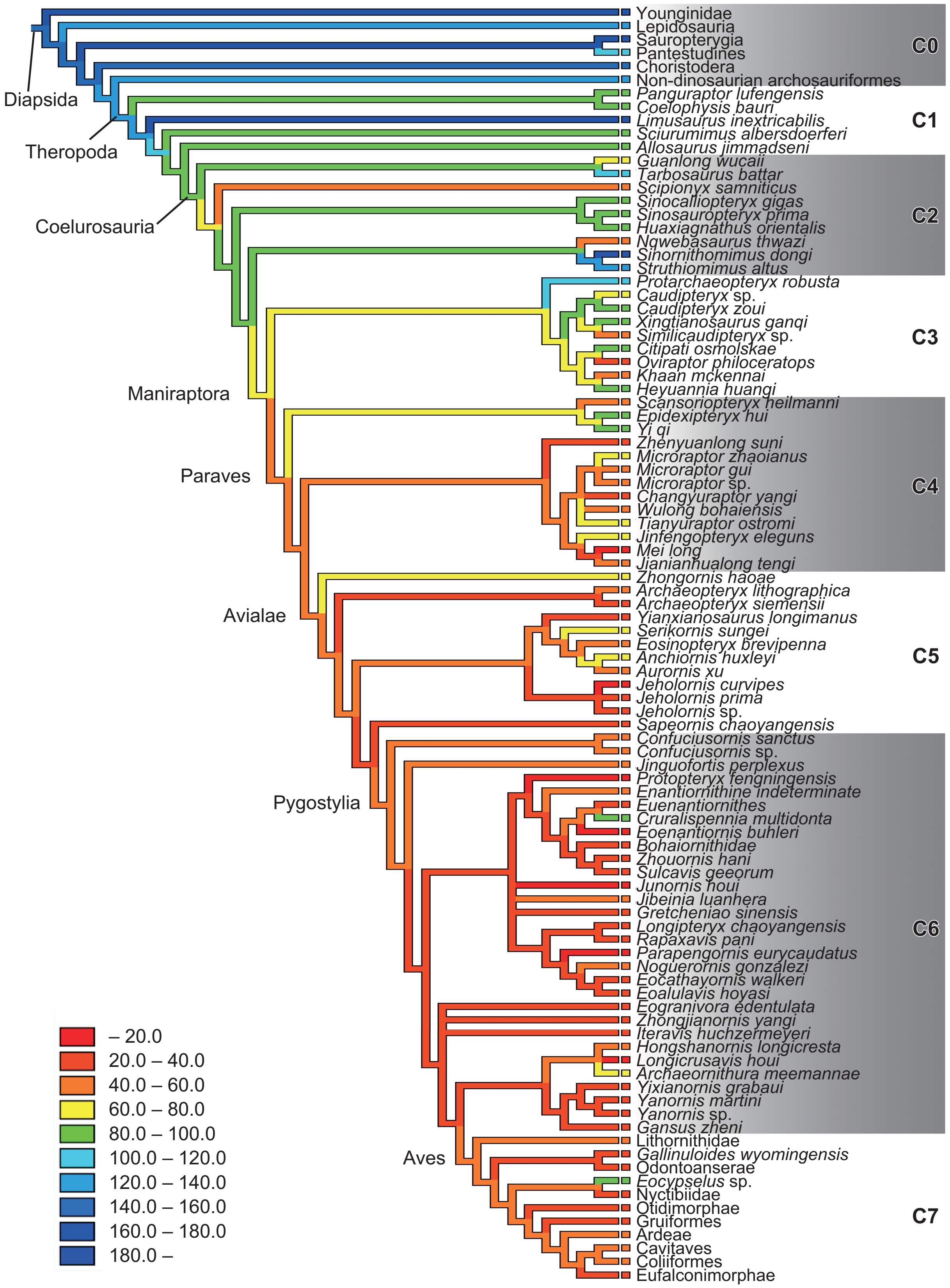

Figure S1. Ancestral state reconstruction of the preserved elbow-joint angles.

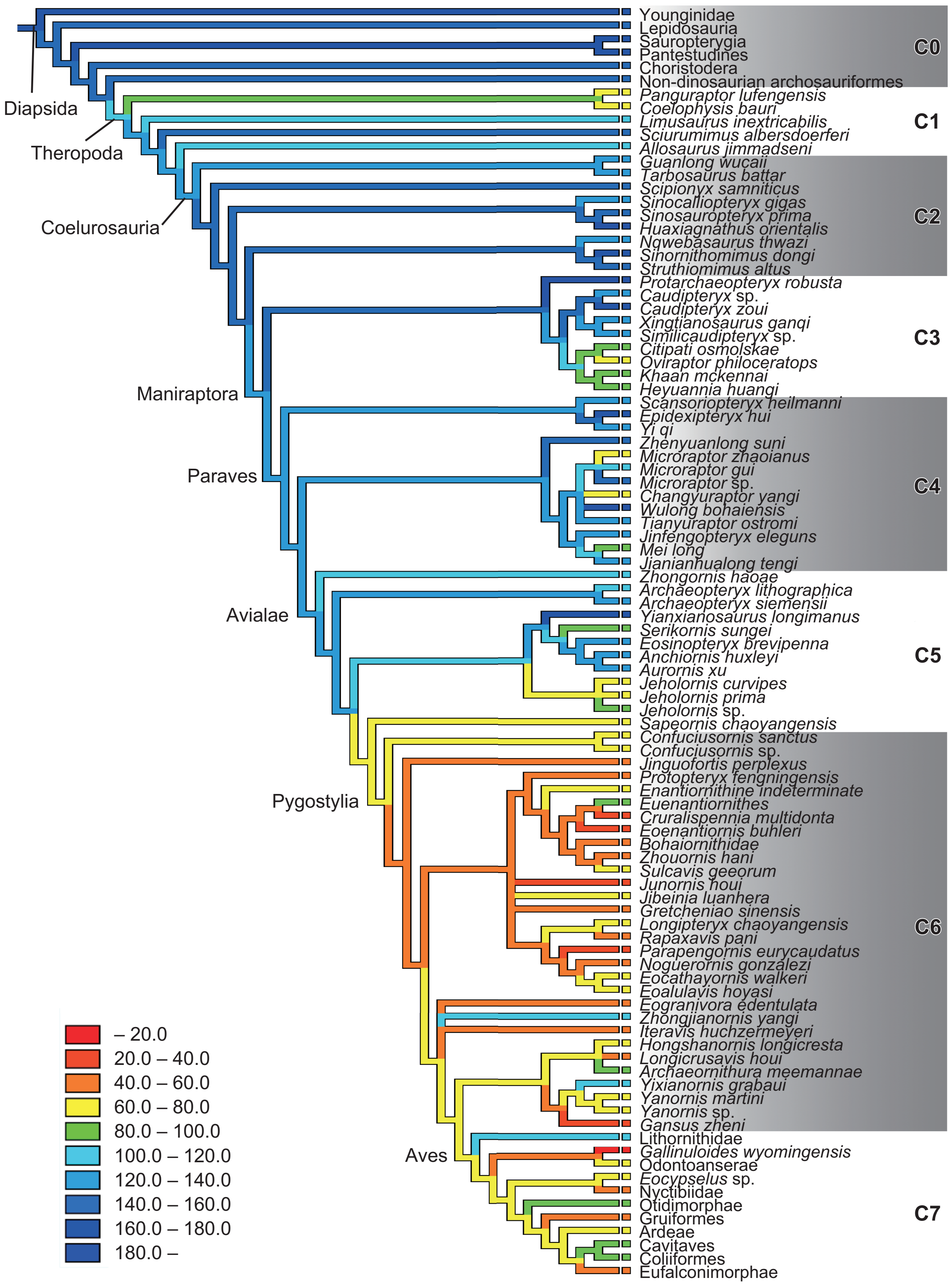

Figure S2. Ancestral state reconstruction of the preserved wrist-joint angles.

Table S1. List of specimens.

Category	Species	Specimen No.	Reference
C0	*Thadeosaurus colcanapi*	MNHN 1908-11-13a	[43]
	Odontochelys semitestacea	IVPP V 13240	[44]
	Chisternon undotum _1	FMNH PR 2451	[43]
	Chisternon undotum _2	WDC-CGR-32	[43]
	Chisternon undotum _3	FMNH PR 2829	[43]
	Baena arenosa	FMNH PR 2391	[43]
	Thalassemys bruntrutana	NKMB Watt18/211	[45]
	Tropidemys seebachi	NKMB Watt09/162	[46]
	Jeholochelys lingyuanensis	PMOL-AR00213	[47]
	Tasbacka danica	MHM-K2	[48]
	Allaeochelys crassesculptata	HLMD-Me 7593	[49]
	Hummelichelys guttata	collection of Fossil Butte National Monument	[43]
	Axestemys byssinus _1	FMNH PR 3039	[43]
	Axestemys byssinus _2	WDC-CGR 111	[43]
	Palaeoamyda messeliana	WDC-C-MG-310	[50]
	Trionyx messelianus	HLMD-Me 8036	[49]
	Eusaurosphargis dalsassoi	PIMUZ A/III 4380	[51]
	Dianopachysaurus dingi	LPV 31365	[52]
	Keichousaurus hui	NMNS-VL191	[53]
	Lariosaurus sp.	LPV 301881	[52]
	Diandongosaurus acutidentatus _1	IVPP V 17761	[52]
	Diandongosaurus acutidentatus _2	WIGM SPC V 1105	[54]
	Megachirella wachtleri	PZO 628	[55]
	Vadasaurus herzogi	AMNH FARB 32768	[56]
	Palaeopleurosaurus sp.	SMNS 81774	[57]
	Derasmosaurus pietraroiae	MPN 541	[33]
	Sphenofontis velserae	SNSB-BSPG 1993 XVIII 4	[58]
	Lacertulus bipes	DNMNH 3/3602	[59]
	Geiseltaliellus longicaudus	HLMD-Me 10207	[49]
	Huehuecuetzpalli mixtecus _1	IGM 4185	[60]
	Huehuecuetzpalli mixtecus _2	IGM 7389	[60]
	Xianglong zhaoi	LPM-000666	[61]
	Daohugou lizard	IVPP V 13747	[62]

	Afariguana avius	FMNH PR 2379	[43]
	Eolacerta robusta	HLMD-Be 146	[49]
	Lacertoidea	JZC Bu1803	[63]
	Ardeosaurus digitatellus	CM 4026	[64]
	Ardeosaurus brevipes	PMU.R58	[65]
	Eichstaettisaurus schroederi	BSPG 1937 I 1	[64]
	Eichstaettisaurus gouldi	MNP 19457	[66]
	Squamata indet.	JZC Bu267	[63]
	Adriosaurus suessi	NHM R2867	[67]
	Primitivus manduriensis	MPUR NS 161	[68]
	Jucaraseps grandipes	LH 18505	[69]
	Yabeinosaurus tenuis _1	IVPP V 12641	[70]
	Yabeinosaurus tenuis _2	IVPP V 13284	[70]
	Daohugou lizard	IVPP V 14386	[71]
**C0**	*Bahndwivici ammoskius*	FMNH PR 2260	[43]
	Saniwa ensidens _1	FMNH PR 2378	[43]
	Saniwa ensidens _2	BHI 1285	[43]
	Cryptolacerta hassiaca	SMF-ME 2604	[72]
	Polyglyphanodon sternbergi	CM 9188	[64]
	Hyphalosaurus baitaigouensis	CAGS-IG-06131	[73]
	Lazarussuchus sp.	BDL 1819	[74]
	Monjurosuchus splendens	GMV 2135	[75]
	Hyphalosaurus lingyuanensis _1	IVPP V 11075	[76]
	Hyphalosaurus lingyuanensis _2	PKUP V 1052	[76]
	Litorosuchus somnii	IVPP V 16978	[77]
	Atoposaurus jowdani	MHNL-15679	[78]
	Alligatorellus beaumonti _1	MNHN 15638	[79]
	Alligatorellus beaumonti _2	MNHN15639	[79]
	Alligatorellus bavaricus	BSPG 1937-I-26	[80]
	Alligatorium depereti	MMG-512	[78]
	Borealosuchus wilsoni	FMNH PR1674	[43]
	Crocodilaemus robustus	NHM 40344	[80]
	Diplocynodon darwini _1	HLMD-Me 5317	[49]
	Diplocynodon darwini _2	HLMD-Me 7500	[49]
	Tsoabichi greenriverensis _1	FMNH PR 1793	[43]
	Tsoabichi greenriverensis _2	FMNH PR 3050	[43]
**C1**	*Panguraptor lufengensis*	LFGT-0103	[81]

**C1**	*Coelophysis bauri*	AMNH FR 7223	[82]
	Limusaurus inextricabilis	IVPP V 15923	[83]
	Sciurumimus albersdoerferi	BMMS BK 11	[84]
	Allosaurus jimmadseni	DINO 11541	[85]
**C2**	*Guanlong wucaii*	IVPP V 14532	[86]
	Tarbosaurus battar	MPC-D 107/7	[87]
	Scipionyx samniticus	SBA-SA 163760	[33]
	Sinocalliopteryx gigas	JMP-V-05-8-01	[88]
	Sinosauropteryx prima _1	NIGP 127586	[89]
	Sinosauropteryx prima _2	NIGP 127587	[30, 89]
	Huaxiagnathus orientalis	CAGS-IG02-301	[90]
	Nqwebasaurus thwazi	AM 6040	[91]
	Sinornithomimus dongi	IVPP V 11797	[92]
	Struthiomimus altus	AMNH 5339	[93]
**C3**	*Protarchaeopteryx robusta*	NGMC 2125	[94]
	Caudipteryx sp.	IVPP V 12430	[29], direct observation
	Caudipteryx zoui _1	NGMC 97-4-A	[94]
	Caudipteryx zoui _2	NGMC 97-9-A	[94]
	Xingtianosaurus ganqi	IVPP V 13390	[40]
	Similicaudipteryx sp. _1	STM4-1	[2]
	Similicaudipteryx sp. _2	STM22-6	[2]
	Citipati osmolskae _1	MPC-D 100/1004	[95]
	Citipati osmolskae _2	MPC-D 100/979	[95]
	Oviraptor philoceratops	AMNH FARB 6517	[95]
	Khaan mckennai _1	MPC-D 100/1127	[96]
	Khaan mckennai _2	MPC-D 100/1002	[96]
	Heyuannia huangi	HYMV1-2	[97]
**C4**	*Scansoriopteryx heilmanni*	CAGS02-IG-gausa-1/DM 607	[98]
	Epidexipteryx hui	IVPP V 15471	[99]
	Yi qi	STM31-2	[41]
	Dromaeosauridae indet.	LPM 0200/0201	[100]
	Dromaeosauridae indet.	NGMC 91-A	[101]
	Zhenyuanlong suni	JPM-0008	[37]
	Microraptor zhaoianus	CAGS-20-7-004	[102]
	Microraptor gui _1	IVPP V 13352	[28], direct observation

C4	*Microraptor gui* _2	IVPP V 17972A	[103]
	Microraptor sp.	STM5-32	[104]
	Changyuraptor yangi	HG B016	[105]
	Wulong bohaiensis	DNHM-D2933	[106]
	Tianyuraptor ostromi	STM1-3	[107]
	Jinfengopteryx eleguns	CAGS-IG-04-0801	[108]
	Mei long	IVPP V 12733	[109]
	Jianianhualong tengi	XHPM 1218	[110]
C5	*Zhongornis haoae*	DNHM D2455	[111]
	Archaeopteryx lithographica	HMN 1880	[112]
	Archaeopteryx siemensii	WDC-CSG-100	[113, 114]
	Yianxianosaurus longimanus	IVPP V 12638	[115]
	Serikornis sungei	PMOL-AB00200	[116]
	Eosinopteryx brevipenna	YFGP-T5197	[117]
	Anchiornis huxleyi _1	LPM-B00169	[118]
	Anchiornis huxleyi _2	YFGP-T5199	[119]
	Aurornis xui	YFGP-T5198	[120]
	Jeholornis curvipes	YFGP-yb2	[119]
	Jeholornis prima _1	BMNHC-PH780	[121]
	Jeholornis prima _2	CDL-02-04-001	[121]
	Jeholornis prima _3	IVPP V 13274	[122]
	Jeholornis prima _4	IVPP V 13353	[123]
	Jeholornis sp. _1	STM2-37	[124]
	Jeholornis sp. _2	STM2-51	[125]
C6	*Sapeornis chaoyangensis* _1	DNHM-D3078	[126]
	Sapeornis chaoyangensis _2	STM15-15	[127]
	Sapeornis chaoyangensis _3	HGM-41HIII0405	[121]
	Sapeornis chaoyangensis _4	DNHM-D2523	[121]
	Confuciusornis sanctus _1	LPM-0228	[121]
	Confuciusornis sanctus _2	LPM-0229	[121]
	Confuciusornis sanctus _3	DNHM-D2859	[121]
	Confuciusornis sanctus _4	BMNHC-PH766	[122]
	Confuciusornis sanctus _5	BMNHC-PH931	[123]
	Confuciusornis sanctus _6	BMNHC-PH987	[121]
	Confuciusornis sanctus _7	LPM-0233	[121]
	Confuciusornis sanctus _8	DNHM-D2151	[121]
	Confuciusornis sanctus _9	NIGP 139379_1	[121]

	Confuciusornis sanctus _10	HGM-41HIII0400	[121]
	Confuciusornis sanctus _11	IVPP V 110304	[128]
	Confuciusornis sanctus _12	IVPP V 10918	[129]
	Confuciusornis sanctus _13	MCFO 0589a	[130]
	Confuciusornis sp.	IVPP V 13156	[131]
	Jinguofortis perplexus	IVPP V 24194	[132]
	Protopteryx fengningensis _1	BMNHC-PH1158A	[121]
	Protopteryx fengningensis _2	BMNHC-PH1060A/B	[121]
	Protopteryx fengningensis _3	IVPP V 1165	[133]
	Enantiornithine indet._1	BMNHC-PH1154A	[121]
	Enantiornithine indet._2	BMNHC-PH1154B	[121]
	Enantiornithine indet._3	BMNHC-PH1156A	[121]
	Enantiornithine indet._4	STM29-8	[121]
	Enantiornithine indet._5	DNHM-D2884/1	[121]
	Enantiornithine indet._6	BMNHC-PH877	[121]
	Enantiornithine indet._7	BMNHC-PH807	[121]
	Enantiornithine indet._8	BMNHC-PH1061B	[121]
**C6**	Enantiornithine indet._9	BMNHC-PH925	[121]
	Enantiornithine indet._10	STM34-1	[134]
	Enantiornithine indet._11	STM34-2	[134]
	Enantiornithine indet._12	STM34-7	[134]
	Euenantiornithes indet.	UFRJ-DG 031 Av	[135]
	Cruralispennia multidonta	IVPP V 21711	[136]
	Eoenantiornis buhleri	IVPP 11537	[137]
	Bohaiornithidae indet.	BMNHC-PH1204	[121]
	Zhouornis hani _1	BMNHC-PH756	[121]
	Zhouornis hani _2	CNUVB-0903	[121]
	Sulcavis geeorum	BMNHC-PH805	[121]
	Junornis houi	BMNHC-PH919A	[121]
	Jibeinia luanhera	uncatalogued	[128]
	Gretcheniao sinensis	BMNHC-PH 829	[138]
	Longipteryx chaoyangensis _1	BMNHC-PH826	[121]
	Longipteryx chaoyangensis _2	HGM-41HIII0319	[121]
	Longipteryx chaoyangensis _3	BMNHC-PH1071	[121]
	Rapaxavis pani	DNHM-D2522	[121]
	Parapengornis eurycaudatus	IVPP V 18687	[139]
	Noguerornis gonzalezi	LP. 1702. P	[129]

	Eocathayornis walker	IVPP V 10916A/B	[121]
	Eoalulavis hoyasi	LH-13500a	[129]
	Eogranivora edentulate	STM35-3	[140]
	Zhongjianornis yangi	IVPP V 15900	[141]
	Iteravis huchzermeyeri _1	AGB5834-1	[142]
	Iteravis huchzermeyeri _2	AGB5834-2	[142]
	Hongshanornis longicresta _1	DNHM-D2945	[143]
	Hongshanornis longicresta _2	DNHM-D2946	[143]
	Hongshanornis longicresta _3	IVPP V 14533B	[144]
	Longicrusavis houi	IVPP V 11309	[121]
	Archaeornithura meemannae	STM7-145	[145]
**C6**	*Yixianornis grabaui* _1	IVPP V 12631	[121]
	Yixianornis grabaui _2	IVPP V 13631	[146]
	Yanornis martini _1	BMNHC-PH1043	[121]
	Yanornis martini _2	XHPM-1205	[121]
	Yanornis martini _3	DNHM-D3069	[121]
	Yanornis martini _4	IVPP V 13358	[147]
	Yanornis martini _5	IVPP V 12558	[148]
	Yanornis sp. _1	STM9-15	[149]
	Yanornis sp. _2	STM9-46	[149]
	Gansus zheni _1	BMNHC-PH1318	[121]
	Gansus zheni _2	BMNHC-PH11392	[121]
	Gansus zheni _3	BMNHC-PH1342	[121]
	Pseudocrypturus cercanaxius	WDC-CGR-108	[43]
	Pseudocrypturus sp.	SMA 0186	[43]
	Gallinuloides wyomingensis _1	WDC-CGR 012	[43]
	Gallinuloides wyomingensis _2	MCZ 2221	[43]
	Salmila robusta	HLMD-Be 161	[49]
	Messelornis nearctica _1	BHI 6298	[43]
	Messelornis nearctica _2	SMF AV406	[43]
**C7**	"waterfowl"	FMNH PA 725	[43]
	Parargornis messelensis	HLMD-Be 193	[49]
	Eocypselus sp.	WDC-CGR-109	[43]
	Paraprefica major	SMNS 81654	[150]
	Paraprefica kelleri _1	SMF-ME 3376	[150]
	Paraprefica kelleri _2	HLMD-Be 164	[49]
	Foro panarium	USNM 336261	[43]

	Masillapodargus longipes	SMNK.PAL.1083	[150]
	Sunbittern?	USNM 336377	[43]
	Limnofregata azygosternon _1	USNM 22753	[43]
	Limnofregata azygosternon _2	FMNH PA 755	[43]
	Plesiocanthus wyomingensis	WDC-2001-CGR-021	[43]
	Eocoracias brachyptera _1	HLMD-Me 10474	[151]
	Eocoracias brachyptera _2	SMNK.PAL.2663	[151]
	Septencoracias morsensis	MGUH.VP 9509	[152]
	Neanis kistneri	USNM 336268	[43]
	Primobucco mcgrewi _1	FMNH PA 724	[43]
**C7**	*Primobucco mcgrewi* _2	FMNH PA 611	[153]
	Primobucco mcgrewi _3	FMNH PA 738	[153]
	Primobucco mcgrewi _4	UWGM 14563	[153]
	Primobucco mcgrewi _5	WSGS U-93–1A	[153]
	Anneavis anneae	BMS E 25337	[43]
	Celericolius acriala	FMNH PA 730	[43]
	Tynskya eocaena	BSPG 1997 I 6	[43]
	Eozygodactylus americanus	FMNH PA 726	[43]
	Passer domesticus	SNHM/ 6105	[154]
	Cyrilavis colburnorum	FMNH PA 754	[43]
	Cyrilavis olsoni	UAM PV 2005.6.196	[43]

Supplementary Table 2. Measurements of the elbow- and wrist-joint angles.

Category	Species	Elbow angle	Wrist angle
	Thadeosaurus colcanapi	172.90	171.76
	Odontochelys semitestacea	103.77	147.61
	Chisternon undotum	112.10	155.83
	Baena arenosa	145.22	256.24
	Thalassemys bruntrutana	94.17	167.38
	Tropidemys seebachi	88.88	172.30
	Jeholochelys lingyuanensis	102.10	179.01
	Tasbacka danica	110.63	169.54
	Allaeochelys crassesculptata	57.34	166.07
	Hummelichelys guttata	149.26	200.62
	Axestemys byssinus	84.50	212.54
	Palaeoamyda messeliana	106.51	132.84
	Trionyx messelianus	80.78	153.30
	Eusaurosphargis dalsassoi	174.34	143.49
	Dianopachysaurus dingi	159.96	154.09
	Keichousaurus hui	174.08	175.36
	Lariosaurus sp.	176.43	170.48
	Diandongosaurus acutidentatus	177.07	166.69
**C0**	*Megachirella wachtleri*	126.85	172.26
	Vadasaurus herzogi	144.00	144.46
	Palaeopleurosaurus	172.88	164.92
	Derasmosaurus pietraroiae	145.34	174.94
	Sphenofontis velserae	121.00	160.25
	Lacertulus bipes	171.33	154.23
	Geiseltaliellus longicaudus	137.59	130.83
	Huehuecuetzpalli mixtecus	118.12	146.48
	Xianglong zhaoi	120.51	155.87
	Daohugou lizard_ IVPP V 13747		
	Afairiguana avius	127.19	157.69
	Eolacerta robusta	89.48	171.61
	Lacertoidea	138.15	154.59
	Ardeosaurus digitatellus	143.44	151.69
	Ardeosaurus brevipes	122.16	153.62
	Eichstaettisaurus schroederi	103.16	157.11

	Eichstaettisaurus gouldi	139.18	167.20
	Squamata	123.23	120.63
	Adriosaurus suessi	121.89	168.47
	Primitivus manduriensis	164.27	164.61
	Jucaraseps grandipes	151.45	172.19
	Yabeinosaurus tenuis	139.07	157.51
	Daohugou lizard_ IVPP V 14386		
	Bahndwivici ammoskius	121.11	174.89
	Saniwa ensidens	126.07	194.99
	Cryptolacerta hassiaca	138.68	108.31
	Polyglyphanodon sternbergi	31.70	157.07
**C0**	*Hyphalosaurus baitaigouensis*	168.08	156.71
	Hyphalosaurus lingyuanensis	146.91	155.88
	Lazarussuchus sp.	176.68	151.37
	Monjurosuchus splendens	143.26	146.95
	Litorosuchus somnii	154.15	177.09
	Atoposaurus jowdani	163.74	169.76
	Alligatorellus beaumonti	143.37	158.69
	Alligatorellus bavaricus	151.89	155.80
	Alligatorium depereti	126.92	158.25
	Borealosuchus wilsoni	100.42	177.26
	Crocodilaemus robustus	87.89	84.32
	Diplocynodon darwini	142.75	125.42
	Tsoabichi greenriverensis	172.48	171.63
	Panguraptor lufengensis	86.07	72.54
	Coelophysis bauri	88.73	74.37
**C1**	*Limusaurus inextricabilis*	166.10	113.55
	Sciurumimus albersdoerferi	80.61	147.20
	Allosaurus jimmadseni	89.60	112.04
	Guanlong wucaii	78.87	136.48
	Tarbosaurus battar	110.85	135.90
	Scipionyx samniticus	49.16	153.70
	Sinocalliopteryx gigas	91.27	121.48
**C2**	*Sinosauropteryx prima*	98.98	150.28
	Huaxiagnathus orientalis	86.16	211.57
	Nqwebasaurus thwazi	53.37	135.24
	Sinornithomimus dongi	183.13	166.03

**C2**	*Struthiomimus altus*	133.49	157.92
	Protarchaeopteryx robusta	108.66	182.75
	Caudipteryx sp.	75.93	129.41
	Caudipteryx zoui	98.38	163.39
	Xingtianosaurus ganqi	84.62	128.14
**C3**	*Similicaudipteryx* sp.	55.22	133.01
	Citipati osmolskae	87.15	99.30
	Oviraptor philoceratops	38.58	72.19
	Khaan mckennai	45.17	89.55
	Heyuannia huangi	89.62	85.29
	Scansoriopteryx heilmanni	50.33	127.12
	Epidexipteryx hui	88.48	169.52
	Yi qi	94.10	120.15
	Dromaeosauridae (BPM 1 3-13)	47.55	61.42
	Dromaeosauridae (NGMC 91-A)	13.32	84.94
	Zhenyuanlong suni	24.83	152.87
	Microraptor zhaoianus	78.92	70.57
**C4**	*Microraptor gui*	46.53	113.73
	Microraptor sp.	43.33	151.12
	Changyuraptor yangi	29.00	79.79
	Wulong bohaiensis	50.82	194.31
	Tianyuraptor ostromi	69.76	121.22
	Jinfengopteryx eleguns	60.09	129.63
	Mei long	15.39	82.45
	Jianianhualong tengi	44.44	120.62
	Zhongornis haoae	64.16	116.63
	Archaeopteryx lithographica	40.68	116.03
	Archaeopteryx siemensii	25.79	130.09
	Yixianosaurus longimanus	34.60	162.51
	Serikornis sungei	68.87	85.70
**C5**	*Eosinopteryx brevipenna*	52.73	131.61
	Anchiornis huxleyi	68.64	139.54
	Aurornis xui	56.82	126.11
	Jeholornis curvipes	9.41	63.03
	Jeholornis prima	35.35	70.26
	Jeholornis sp.	35.33	80.89
**C6**	*Sapeornis chaoyangensis*	24.44	70.04

	Confuciusornis sanctus	44.87	63.80
	Confuciusornis sp.	51.93	68.00
	Jinguofortis perplexus	46.70	46.33
	Protopteryx fengningensis	12.02	46.05
	Enantiornithine indeterminate	41.84	65.93
	Euenantiornithes	39.50	88.68
	Cruralispennia multidonta	84.31	30.43
	Eoenantiornis buhleri	11.36	36.98
	Bohaiornithidae	32.43	50.82
	Zhouornis hani	20.75	48.30
	Sulcavis geeorum	39.50	62.21
	Junornis houi	53.00	64.23
	Jibeinia luanhera	18.77	36.30
	Gretcheniao sinensis	30.24	42.62
	Longipteryx chaoyangensis	28.76	76.27
**C6**	*Rapaxavis pani*	34.39	43.19
	Parapengornis eurycaudatus	11.90	31.54
	Noguerornis gonzalezi	49.37	59.36
	Eocathayornis walkeri	31.73	63.95
	Eoalulavis hoyasi	31.52	74.76
	Eogranivora edentulata	29.00	52.59
	Zhongjianornis yangi	27.46	115.98
	Iteravis huchzermeyeri	34.01	50.11
	Hongshanornis longicresta	40.10	67.77
	Longicrusavis houi	14.79	52.94
	Archaeornithura meemannae	63.01	84.88
	Yixianornis grabaui	28.17	117.98
	Yanornis martini	30.20	71.29
	Yanornis sp.	29.26	76.02
	Gansus zheni	29.71	29.35
	Pseudocrypturus cercanaxius	17.92	47.33
	Pseudocrypturus sp.	91.14	159.88
	Gallinuloides wyomingensis	29.92	15.86
**C7**	*Salmila robusta*	22.97	42.97
	Messelornis nearctica	33.18	60.17
	undescribed waterfowl	29.11	64.87
	Parargornis messelensis	45.75	85.57

	Eocypselus sp.	92.00	68.81
	Paraprefica major	13.78	64.02
	Paraprefica kelleri	31.87	54.56
	Foro panarium	20.32	64.14
	Masillapodargus longipes	39.40	98.16
	undescribed (possible sunbittern)	35.98	90.82
	Limnofregata azygosternon	53.90	65.13
	Plesiocanthus wyomingensis	75.25	123.46
**C7**	*Eocoracias brachyptera*	24.12	76.16
	Septencoracias morsensis	38.12	86.58
	Neanis kistneri	55.14	86.48
	Primobucco mcgrewi	30.03	62.85
	Anneavis anneae	51.73	78.91
	Celericolius acriala	81.66	110.71
	Tynskya eocaena	24.69	63.36
	Eozygodactylus americanus	17.36	41.29
	Passer domesticus	27.47	45.56
	Cyrilavis colburnorum	30.57	46.06
	Cyrilavis olsoni	30.72	52.76

List of Institutional Abbreviations

AGB, Anhui Geological Museum, Hefei, China
AM, Albany Museum, Grahamstown, South Africa
AMNH, American Museum of Natural History, New York City, USA
BDL, Musée de Paléontologie de menat BDL (Bord Du Lac), Puy de Dôme, France
BHI, Black Hills Institute of Geological Research, Hill City, USA
BMMS, Bürgermeister Müller Museum Solnhofen, Solnhofen, Germany
BMNHC, Beijing Museum of Natural History, Beijing, China
BMS, Buffalo Museum of Science, Buffalo, USA
BSPG, Bayerische Staatssammlung für Paläontologie und Geologie, Munich, Germany
CAGS, Chinese Academy of Geological Sciences, Beijing, China
CDL, Dinosaurland, Changzhou, China
CM, Carnegie Museum of Natural History, Pittsburgh, USA
CNU Capital Normal University, Beijing, China
DINO, Dinosaur National Monument, Utah, USA
DNHM, Dalian Natural History Museum, Dalian, China
DNMNH, Ditsong National Museum of Natural History (formaly TM, Transvaal Museum), Pretoria, South Africa
FMNH, Field Museum of Natural History, Chicago, USA
GMV, National Geological Museum of China, Beijing, China
HG, Paleontological Center, Bohai University, Jinzhou City, China
HGM, Henan Geological Museum, Zhengzhou, Henan, China
HLMD, Hessisches Landesmuseum Darmstadt, Darmstadt, Germany
HMN, Museum für Naturkunde Berlin, Berlin, Germany (formerly Humboldt Museum für Naturkunde)
HYMV, Heyuan Museum, Guangdong, China
IGM, Instituto de Geología, Universidad Nacional Autónoma de México, México
IVPP, Institute of Vertebrate Paleontology and Paleoanthropology, Beijing, China
JZC, James Zigras Collection
JZMP, Jinzhou Museum of Paleontology, Jinzhou, China
LFGT, Bureau of Land and Resources of Lufeng County, Lufeng, China
LH, Las Hoyas Collection, Museo de Cuenca, Cuenca, Spain
LP, La Pedrera Collection, Institut d'Estudis Ilerdencs, Lleida, Spain
LPM, Liaoning Paleontological Museum, Beipiao, China
LPV, Chengdu Center of China Geological Survey, Chengdu, China
MCFO, CosmoCaixa, Barcelona, Spain
MCZ, Museum of Comparative Zoology, Harvard University, Cambridge, USA
MMG, Museo Martorell de Geologia, Barcelona, Spain
MFSN, Museo Friulano di Storia Naturale, Udine, Italia
MGUH, Museum Geologicum Universitatis Hafniensis (Geological Museum, University of Copenhagen), Copenhagen, Denmark
MHM, Moclay Museum, Nykobing Mors, Denmark
MHNL, Musée Guimet d'Histoire Naturelle, Lyon, France
MNHN, Muséum National d'Histoire Naturelle, Paris, France

MNP, Museo di Paleontologia, Napoli, Italia
MPC, Mongolian Paleontological Center, Mongolian Academy of Sciences, Ulaanbaatar, Mongolia
MPN, Museo di Paleontologia, Napoli, Italia
MPUR, Museo Paleontologico dell'Università di Roma, Lazio, Italy
NGMC, National Geological Museum of China, Beijing, China
NHM, Natural History Museum, London, UK
NIGP, Nanjing Institute of Geology and Paleontology, Nanjing, China
NKMB, Naturkunde-Museum Bamberg, Bamberg, Germany
NMNS, National Museum of Natural Science, Taichung, Taiwan
PIMUZ, University of Zurich Paleontology Museum, Zurich, Switzerland
PKUP, Peking University Paleontological Collections, Beijing, China
PMOL, Paleontological Museum of Liaoning, Shenyang, China
PMU, Paleontological Museum of Uppsala University, Uppsala, Sweden
PZO, Museo Archeologico dell'Alto Adige, Bolzano (Bozen), Italy
SBA-SA, Soprintendenza per i Beni Archeologici di Salerno Avellino Benevento e Caserta, Salerno, Italia
SMA, Sauriermuseum, Aathal, Switzerland
SMF, Senckenberg Forschungsinstitut und Naturmuseum, Frankfurt am Main, Germany
SMNK, Staatliches Museum für Naturkunde Karlsruhe, Karlsruhe, Germany
SMNS, Staatliches Museum für Naturkunde Stuttgart, Stuttgart, Germany
SNHM, Staatliches Naturhistorisches Museum Braunschweig, Braunschweig, Germany
SNSB-BSPG, Staatliche Naturwissenschaftliche Sammlungen Bayerns-Bayerische Staatssammlung für Paläontologie und Geologie, Munich, Germany
STM, Shandong Tianyu Museum of Nature, Pingyi, China
UAM, University of Alaska Museum, Fairbanks, USA
UFRJ-DG, Universidade Federal do Rio de Janeiro, Departamento de Geologia, Rio de Janeiro, Brazil
USNM, National Museum of Natural History, Smithsonian Institution, Washington, D.C., USA
UWGM, University of Wisconsin-Madison, Madison, USA
WDC, Wyoming Dinosaur Center, Thermopolis, USA
WIGM, Wuhan Institute of Geology and Mineral Resources, Hubei, China
WSGS, Wyoming State Geological Survey, Laramie, USA
XHPM, Xinghai Paleontological Museum, Dalian, China
YFGP, Yizhou Fossil & Geology Park, Panjin, China

Supplementary References

43. Grande L. The Lost World of Fossil Lake. Snap Shots from Deep Time. Chicago: University of Chicago Press; 2013.
44. Li C, Wu XC, Rieppel O, Wang LT, Zhao LJ. An ancestral turtle from the Late Triassic of southwestern China. *Nature*. 2008;456:497–501.
45. Joyce WG, Mäuser M, Evers SW. Two turtles with soft tissue preservation from the platy limestones of Germany provide evidence for marine flipper adaptations in Late Jurassic thalassochelydians. *PLoS One*. 2021;16:e0252355.
46. Joyce WG, Mäuser M. New material of named fossil turtles from the Late Jurassic (late Kimmeridgian) of Wattendorf, Germany. *PLoS One*. 2020;15:e0233483.
47. Shao S, Li L, Yang Y, Zhou CF. Hyperphalangy in a new sinemydid turtle from the Early Cretaceous Jehol Biota. *PeerJ*. 2018;6:e5371.
48. Karl H-V, Madsen H. *Tasbacka danica* n. sp., a new Eocene marine turtle of Denmark (Testudines: Chelonioidea). *Stud Palaeocheloniologica*. 2012;4:193–204.
49. Gruber G, Micklich N. Messel: Treasures of the Eocene. Darmstadt : Hess Landesmuseum. 2011.
50. Cadena E. *Palaeoamyda messeliana* nov. comb. (Testudines, Pan-Trionychidae) from the Eocene Messel Pit and Geiseltal localities, Germany, taxonomic and phylogenetic insights. *PeerJ*. 2016;4:e2647.
51. Scheyer TM, Neenan JM, Bodogan T, Furrer H, Obrist C, Plamondon M. A new, exceptionally preserved juvenile specimen of *Eusaurosphargis dalsassoi* (Diapsida) and implications for Mesozoic marine diapsid phylogeny. *Sci Rep*. 2017;7:4406.
52. Liu J, Hu SX, Rieppel O, Jiang DY, Benton MJ, Kelley NP, et al. A gigantic nothosaur (Reptilia: Sauropterygia) from the Middle Triassic of SW China and its implication for the Triassic biotic recovery. *Sci Rep*. 2014;4:7142.
53. Cheng YN, Wu XC, Ji Q. Triassic marine reptiles gave birth to live young. *Nature*. 2004;432:383–6.
54. Liu Q, Yang T, Cheng L, Benton MJ, Moon BC, Yan C, et al. An injured pachypleurosaur (Diapsida: Sauropterygia) from the Middle Triassic Luoping Biota indicating predation pressure in the Mesozoic. *Sci Rep*. 2021;11:21818.
55. Simões TR, Caldwell MW, Tałanda M, Bernardi M, Palci A, Vernygora O, et al. The origin of squamates revealed by a Middle Triassic lizard from the Italian Alps. *Nature*. 2018;557:706–9.
56. Bever GS, Norell M. A new rhynchocephalian (Reptilia: Lepidosauria) from the Late Jurassic of Solnhofen (Germany) and the origin of the marine Pleurosauridae. *R Soc Open Sci*. 2017;4:170570.
57. Klein N, Scheyer TM. Microanatomy and life history in *Palaeopleurosaurus* (Rhynchocephalia: Pleurosauridae) from the Early Jurassic of Germany. *Naturwissenschaften*. 2017;104:4.
58. Villa A, Montie R, Röper M, Rothgaenger M, Rauhut OWM. *Sphenofontis velserae* gen. et sp. nov., a new rhynchocephalian from the Late Jurassic of Brunn (Solnhofen Archipelago, Southern Germany). *PeerJ*. 2021;9:e11363.
59. Carroll RL, Thompson P. A bipedal lizardlike reptile from the Karroo. *J Paleontol*. 1982;56:1–10.
60. Reynoso VH. *Huehuecuetzpalli mixtecus* gen. et sp. nov: a basal squamate (Reptilia) from the Early Cretaceous of Tepexi de Rodriguez, Central México. *Philos Trans R Soc B Biol Sci*. 1998;353:477–500.

61. Li PP, Gao KQ, Hou LH, Xu X. A gliding lizard from the Early Cretaceous of China. *Proc Natl Acad Sci U S A*. 2007;104:5507–09.
62. Evans SE, Wang Y. A long-limbed lizard from the Upper Jurassic/Lower Cretaceous of Daohugou, Ningcheng, Nei Mongol, China. *Vertebr Palasiat*. 2009;47:21–34.
63. Daza JD, Stanley EL, Wagner P, Bauer AM, Grimaldi DA. Mid-Cretaceous amber fossils illuminate the past diversity of tropical lizards. *Sci Adv*. 2016;2:e1501080.
64. Simões TR, Funston GF, Vafaeian B, Nydam RL, Doschak MR, Caldwell MW. Reacquisition of the lower temporal bar in sexually dimorphic fossil lizards provides a rare case of convergent evolution. *Sci Rep*. 2016;6:24087.
65. Mateer NJ. Osteology of the Jurassic lizard *Ardeosaurus brevipes* (Meyer). *Palaeontology*. 1982;25:449–61.
66. Evans SE, Raia P, Barbera C. New lizards and rhynchocephalians from the Lower Cretaceous of southern Italy. *Acta Palaeontol Pol*. 2004;49:393–408.
67. Lee MSY, Caldwell MW. *Adriosaurus* and the affinities of mosasaurs, dolichosaurs, and snakes. J Paleontol. 2000;74:915–37.
68. Paparella I, Palci A, Nicosia U, Caldwell MW. A new fossil marine lizard with soft tissues from the Late Cretaceous of southern Italy. *R Soc Open Sc*i. 2018;5:172411.
69. Bolet A, Evans SE. A tiny lizard (Lepidosauria, Squamata) from the Lower Cretaceous of Spain. *Palaeontology*. 2012;55:491–500.
70. Evans SE, Wang Y, Li C. The early cretaceous lizard genus *Yabeinosaurus* from China: resolving an enigma. *J Syst Palaeontol*. 2005;3:319–335.
71. Evans SE, Wang Y. A juvenile lizard specimen with well-preserved skin impressions from the Upper Jurassic/Lower Cretaceous of Daohugou, Inner Mongolia, China. *Naturwissenschaften*. 2007;94:431–9.
72. Müller J, Hipsley CA, Head JJ, Kardjilov N, Hilger A, Wuttke M, et al. Eocene lizard from Germany reveals amphisbaenian origins. *Nature*. 2011;473:364–7.
73. Ji Q, Wu XC, Cheng YN. Cretaceous choristoderan reptiles gave birth to live young. *Naturwissenschaften*. 2010;97:423–8.
74. Matsumoto R, Buffetaut E, Escuillie F. New material of the choristodere *Lazarussuchus* (Diapsida, Choristodera) from the Paleocene of France. *J Vertebr Paleontol*. 2013;33:319–39.
75. Gao KQ, Evans S, Qiang J, Norell M, Shu'An J. Exceptional fossil material of a semi-aquatic reptile from China: The resolution of an enigma. *J Vertebr Paleontol*. 2000;20:417–21.
76. Gao KQ, Ksepka DT. Osteology and taxonomic revision of *Hyphalosaurus* (Diapsida: Choristodera) from the Lower Cretaceous of Liaoning, China. *J Anat.* 2008;212:747–768.
77. Li C, Wu XC, Zhao LJ, Nesbitt SJ, Stocker MR, Wang LT. A new armored archosauriform (Diapsida: Archosauromorpha) from the marine Middle Triassic of China, with implications for the diverse life styles of archosauriforms prior to the diversification of Archosauria. *Naturwissenschaften*. 2016;103:95.
78. Buscalioni AD, Sanz JL. Phylogenetic relationships of the atoposauridae (Archosauria, Crocodylomorpha). *Hist Biol*. 1988;1:244–54.
79. Tennant JP, Mannion PD. Revision of the Late Jurassic crocodyliform *Alligatorellus*, and evidence for allopatric speciation driving high diversity in western European atoposaurids. *PeerJ*. 2014;2:e599.

80. Young MT, Bell MA, de Andrade MB, Brusatte SL. Body size estimation and evolution in metriorhynchid crocodylomorphs: implications for species diversification and niche partitioning. *Zool J Linn Soc*. 2011;163:1199–216.
81. You HL, Azuma Y, Wang T, Wang YM, Dong ZM. The first well-preserved coelophysoid theropod dinosaur from Asia. *Zootaxa*. 2014;3873:233–49.
82. Nesbitt SJ, Turner AH, Erickson GM, Norell MA. Prey choice and cannibalistic behaviour in the theropod *Coelophysis*. *Biol Lett*. 2006;2:611–14.
83. Xu X, Clark JM, Mo J, Choiniere J, Forster CA, Erickson GM, et al. A Jurassic ceratosaur from China helps clarify avian digital homologies. *Nature*. 2009;459:940–4.
84. Rauhut OWM, Foth C, Tischlinger H, Norell MA. Exceptionally preserved juvenile megalosauroid theropod dinosaur with filamentous integument from the Late Jurassic of Germany. *Proc Natl Acad Sci U S A*. 2012;109:11746–51.
85. Chure DJ, Loewen MA. Cranial anatomy of *Allosaurus jimmadseni*, a new species from the lower part of the Morrison Formation (Upper Jurassic) of Western North America. *PeerJ*. 2020;1:7803.
86. Xu X, Clark JM, Forster CA, Norell MA, Erickson GM, Eberth DA, et al. A basal tyrannosauroid dinosaur from the Late Jurassic of China. *Nature*. 2006;439:715–8.
87. Tsuihiji T, Watabe M, Tsogtbaatar K, Tsubamoto T, Barsbold R, Suzuki S, et al. Cranial osteology of a juvenile specimen of *Tarbosaurus bataar* (Theropoda, Tyrannosauridae) from the Nemegt Formation (Upper Cretaceous) of Bugin Tsav, Mongolia. *J Vertebr Paleontol*. 2011;31:497–517.
88. Ji S, Ji Q, Lu J, Yuan CX. A new giant compsognathid dinosaur with long filamentous integuments from Lower Cretaceous of Northeastern China. *Acta Geol Sin Ed*. 2007;81:8–15.
89. Currie PJ, Chen PJ. Anatomy of *Sinosauropteryx prima* from Liaoning, northeastern China. *Can J Earth Sci*. 2001;38:1705–27.
90. Hwang SH, Norell MA, Qiang J, Keqin G. A large Compsognathid from the early cretaceous Yixian formation of China. *J Syst Palaeontol*. 2004;2:13–30.
91. De Klerk WJ, Forster CA, Ross CF, Sampson SD, Chinsamy A. A new coelurosaurian dinosaur from the early cretaceous of south africa. *J Vertebr Paleontol*. 2000;20:324–32.
92. Kobayashi Y, Lü JC. A new ornithomimid dinosaur with gregarious habits from the Late Cretaceous of China. *Acta Palaeontol Pol*. 2003;48:235–59.
93. Osborn HF. Skeletal adaptations of *Ornitholestes*, *Struthiomimus*, *Tyrannosaurus*. *Bull Am Museum Nat Hist*. 1916;35:733–71.
94. Qiang J, Currie PJ, Norell MA, Shu-An J. Two feathered dinosaurs from northeastern china. *Nature*. 1998;393:753–61.
95. Norell MA, Balanoff AM, Barta DE, Erickson GM. A second specimen of *Citipati osmolskae* associated with a nest of eggs from Ukhaa Tolgod, Omnogov Aimag, Mongolia. *Am Museum Novit*. 2018;3899:1–44.
96. Clark JM, Norell MA, Barsbold R. Two new oviraptorids (Theropoda: Oviraptorosauria), Upper Cretaceous Djadokhta Formation, Ukhaa Tolgod, Mongolia. *J Vertebr Paleontol*. 2001;21:209–13.
97. Lü J. A new oviraptorosaurid (Theropoda: Oviraptorosauria) from the Late Cretaceous of Southern China. *J Vertebr Paleontol*. 2003;22:871–75.
98. Czerkas SA, Feduccia A. Jurassic archosaur is a non-dinosaurian bird. *J Ornithol*. 2014;155:841–51.

99. Zhang F, Zhou Z, Xu X, Wang X, Sullivan C. A bizarre Jurassic maniraptoran from China with elongate ribbon-like feathers. *Nature*. 2008;455:1105–8.

100. Norell M, Ji Q, Gao K, Yuan C, Zhao Y, Wang L. 'Modern' feathers on a non-avian dinosaur. *Nature*. 2002;416:36–7.

101. Ji Q, Norell MA, Gao KQ, Ji SA, Ren D. The distribution of integumentary structures in a feathered dinosaur. Nature. 2001;410:1084–8.

102. Hwang SH, Norell MA, Qiang J, Keqin G. New Specimens of *Microraptor zhaoianus* (Theropoda: Dromaeosauridae) from Northeastern China. A*m Museum Novit*. 2002;3381:420–8.

103. O'Connor J, Zhou Z, Xu X. Additional specimen of *Microraptor* provides unique evidence of dinosaurs preying on birds. *Proc Natl Acad Sci U S A*. 2011;108:19662–19665.

104. O'Connor J, Zheng X, Dong L, Wang X, Wang Y, Zhang X, et al. *Microraptor* with ingested lizard suggests mon-specialized digestive function. *Curr Biol*. 2019;29:2423–2429.

105. Han G, Chiappe LM, Ji SA, Habib M, Turner AH, Chinsamy A, et al. A new raptorial dinosaur with exceptionally long feathering provides insights into dromaeosaurid flight performance. *Nat Commun*. 2014;5:4382.

106. Poust AW, Gao C, Varricchio DJ, Wu J, Zhang F. A new microraptorine theropod from the Jehol Biota and growth in early dromaeosaurids. *Anat Rec*. 2020;303:963–87.

107. Zheng X, Xu X, You H, Zhao Q, Dong Z. A short-armed dromaeosaurid from the Jehol group of China with implications for early dromaeosaurid evolution. *Proc R Soc B Biol Sci*. 2010;277:211–7.

108. Shu'an J, Qiang J. *Jinfengopteryx* Compared to *Archaeopteryx*, with comments on the mosaic evolution of long-tailed avialan birds. A*cta Geol Sin*. 2007;81:337–43.

109. Xu X, Norell MA. A new troodontid dinosaur from China with avian-like sleeping posture. *Nature*. 2004;431:838–41.

110. Xu X, Currie P, Pittman M, Xing L, Meng Q, Lü J, et al. Mosaic evolution in an asymmetrically feathered troodontid dinosaur with transitional features. *Nat Commun.* 2017;8:14972.

111. Gao C, Chiappe LM, Meng Q, O'Connor JK, Wang X, Cheng X, et al. A new basal lineage of early cretaceous birds from China and its implications on the evolution of the avian tail. *Palaeontology*. 2008;51:775–91.

112. Carney RM, Vinther J, Shawkey MD, D'Alba L, Ackermann J. New evidence on the colour and nature of the isolated *Archaeopteryx* feather. *Nat Commun*. 2012;3:637.

113. Mayr G, Pohl B, Peters DS. Paleontology: A well-preserved *Archaeoptryx* specimen with theropod features. *Science*. 2005;310:1483–6.

114. Mayr G, Pohl B, Hartman S, Peters DS. The tenth skeletal specimen of *Archaeopteryx*. *Zool J Linn Soc*. 2007;149:97–116.

115. Xu X, Wang XL. A new maniraptoran dinosaur from the Early Cretaceous Yixian Formation of western Liaoning. *Vertebr Palasiat*. 2003;41:195–202.

116. Lefèvre U, Cau A, Cincotta A, Hu D, Chinsamy A, Escuillié F, et al. A new Jurassic theropod from China documents a transitional step in the macrostructure of feathers. *Naturwissenschaften*. 2017;104:74.

117. Godefroit P, Demuynck H, Dyke G, Hu D, Escuillié F, Claeys P. Reduced plumage and flight ability of a new Jurassic paravian theropod from China. *Nat Commun*. 2013;4:1394.

118. Hu D, Hou L, Zhang L, Xu X. A pre-*Archaeopteryx* troodontid theropod from China with long feathers on the metatarsus. *Nature*. 2009;461:640–3.

119. Prondvai E, Godefroit P, Adriaens D, Hu DY. Intraskeletal histovariability, allometric growth patterns, and their functional implications in bird-like dinosaurs. *Sci Rep*. 2018;8:258.
120. Godefroit P, Cau A, Dong-Yu H, Escuillié F, Wenhao W, Dyke G. A Jurassic avialan dinosaur from China resolves the early phylogenetic history of birds. *Nature*. 2013;498:359–62.
121. Chiappe LM, Qingjin M. Birds of Stone: Chinese Avian Fossils from the Age of Dinosaurs. Baltimore: Johns Hopkins University Press. 2016.
122. Zhou Z, Zhang F. A long-tailed, seed-eating bird from the Early Cretaceous of China. *Nature*. 2002;418:405–9.
123. Zhou Z, Zhang F. *Jeholornis* compared to *Archaeopteryx*, with a new understanding of the earliest avian evolution. *Naturwissenschaften*. 2003;90:220–5.
124. O'Connor J, Wang X, Sullivan C, Zheng X, Tubaro P, Zhang X, et al. Unique caudal plumage of *Jeholornis* and complex tail evolution in early birds. *Proc Natl Acad Sci U S A*. 2013;110:17404–8.
125. Zheng X, O'Connor J, Huchzermeyer F, Wang X, Wang Y, Wang M, et al. Preservation of ovarian follicles reveals early evolution of avian reproductive behaviour. *Nature*. 2013;495:507–11.
126. Gao C, Chiappe LM, Zhang F, Pomeroy DL, Shen C, Chinsamy A, et al. A subadult specimen of the Early Cretaceous bird *Sapeornis chaoyangensis* and a taxonomic reassessment of sapeornithids. *J Vertebr Paleontol*. 2012;32:1103–12.
127. Zheng X, Martin LD, Zhou Z, Burnham DA, Zhang F, Miao D. Fossil evidence of avian crops from the Early Cretaceous of China. *Proc Natl Acad Sci U S A*. 2011;108:15904–7.
128. Hou L. Mesozoic birds of China. Taichung: Phoenix Valley Provincial Aviary of Taiwan. 2001.
129. Chiappe LM, Witmer LM, editors. Mesozoic Birds: Above the Heads of Dinosaurs. Berkeley, University of California Press. 2002.
130. Chinsamy A, Marugán-Lobón J, Serrano FJ, Chiappe L. Osteohistology and life history of the basal pygostylian, *Confuciusornis sanctus*. *Anat Rec*. 2020;303:949–62.
131. Falk AR, Kaye TG, Zhou Z, Burnham DA. Laser fluorescence illuminates the soft tissue and life habits of the early cretaceous bird *Confuciusornis*. *PLoS One*. 2016;11:e0167284.
132. Wang M, Stidham TA, Zhou Z. A new clade of basal Early Cretaceous pygostylian birds and developmental plasticity of the avian shoulder girdle. *Proc Natl Acad Sci U S A*. 2018;115:10708–13.
133. Chiappe LM, Di L, Serrano FJ, Yuguang Z, Meng Q. Anatomy and flight performance of the early enantiornithine bird *Protopteryx fengningensis*: information from new specimens of the Early Cretaceous Huajiying Formation of China. *Anat Rec*. 2020;303:716–31.
134. Zheng X, Wang X, O’Connor J, Zhou Z. Insight into the early evolution of the avian sternum from juvenile enantiornithines. *Nat Commun*. 2012;3:1116.
135. De Souza Carvalho I, Novas FE, Agnolín FL, Isasi MP, Freitas FI, Andrade JA. A Mesozoic bird from Gondwana preserving feathers. *Nat Commun*. 2015;6:7141.
136. Wang M, O'Connor JK, Pan Y, Zhou Z. A bizarre Early Cretaceous enantiornithine bird with unique crural feathers and an ornithuromorph plough-shaped pygostyle. *Nat Commun*. 2017;8:14141.
137. O'Connor JK, Chiappe LM, Chuong CM, Bottjer DJ, You H. Homology and potential cellular and molecular mechanisms for the development of unique feather morphologies in early birds. *Geosci*. 2012;2:157–77.

138. Chiappe LM, Qingjin M, Serrano F, Sigurdsen T, Min W, Bell A, et al. New *Bohaiornis*-like bird from the Early Cretaceous of China: enantiornithine interrelationships and flight performance. *PeerJ*. 2019;7:e7846.
139. Hu H, O'Connor JK, Zhou Z. A new species of Pengornithidae (Aves: Enantiornithes) from the Lower Cretaceous of China suggests a specialized scansorial habitat previously unknown in early birds. *PLoS One*. 2015;10:e0126791.
140. Zheng X, O'Connor JK, Wang X, Wang Y, Zhou Z. Reinterpretation of a previously described Jehol bird clarifies early trophic evolution in the Ornithuromorpha. *Proc R Soc B Biol Sci*. 2018;285:20172494.
141. Zhou Z, Zhang F, Li Z. A new Lower Cretaceous bird from China and tooth reduction in early avian evolution. *Proc R Soc B Biol Sci*. 2010;277:219–227.
142. Wang X, Huang J, Hu Y, Liu X, Peteya J, Clarke JA. The earliest evidence for a supraorbital salt gland in dinosaurs in new Early Cretaceous ornithurines. *Sci Rep*. 2018;8:3969.
143. Chiappe LM, Bo Z, O'Connor JK, Chunling G, Xuri W, Habib M, et al. A new specimen of the early Cretaceous bird *Hongshanornis longicresta*: Insights into the aerodynamics and diet of a basal ornithuromorph. *PeerJ*. 2014;2:e234.
144. Zhou Z, Zhang F. Discovery of an ornithurine bird and its implication for Early Cretaceous avian radiation. *Proc Natl Acad Sci U S A*. 2005;102:18998–9002.
145. Wang M, Zheng X, O’Connor JK, Lloyd GT, Wang X, Wang Y, et al. The oldest record of ornithuromorpha from the early cretaceous of China. *Nat Commun*. 2015;6:6987.
146. Clarke JA, Zhou Z, Zhang F. Insight into the evolution of avian flight from a new clade of Early Cretaceous ornithurines from China and the morphology of *Yixianornis grabaui*. *J Anat*. 2006;208:287–308.
147. Zhou Z, Clarke J, Zhang F, Wings O. Gastroliths in *Yanornis*: an indication of the earliest radical diet-switching and gizzard plasticity in the lineage leading to living birds? *Naturwissenschaften*. 2004;91:571–4.
148. Zhou Z, Clarke JA, Zhang F. *Archaeoraptor*’s better half. *Nature*. 2002;420:285.
149. Zheng X, O'Connor JK, Huchzermeyer F, Wang X, Wang Y, Zhang X, et al. New specimens of *Yanornis* indicate a piscivorous diet and modern alimentary canal. *PLoS One*. 2014;9:e95036.
150. Mayr G. Caprimulgiform birds from the Middle Eocene of Messel (Hessen, Germany). *J Vertebr Paleontol*. 1999;19:521–32.
151. Mayr G, Mourer-Chauviré C. Rollers (Aves: Coraciiformes s.s.) from the Middle Eocene of Messel (Germany) and the Upper Eocene of the Quercy (France). *J Vertebr Paleontol*. 2000;20:533–46.
152. Bourdon E, Kristoffersen A V., Bonde N. A roller-like bird (Coracii) from the Early Eocene of Denmark. *Sci Rep*. 2016;6:34050.
153. Ksepka DT, Clarke JA. *Primobucco mcgrewi* (Aves: Coracii) from the Eocene Green River Formation: New anatomical data from the earliest constrained record of stem rollers. *J Vertebr Paleontol*. 2010;30:215–25.
154. Tanaka G, Zhou B, Zhang Y, Siveter DJ, Parker AR. Rods and cones in an enantiornithine bird eye from the Early Cretaceous Jehol Biota. *Heliyon*. 2017;3:e00479.
